# High-Density Genomic Characterization of Native Croatian Sheep Breeds

**DOI:** 10.3389/fgene.2022.940736

**Published:** 2022-07-15

**Authors:** Ivana Drzaic, Ino Curik, Boris Lukic, Mario Shihabi, Meng-Hua Li, Juha Kantanen, Salvatore Mastrangelo, Elena Ciani, Johannes A. Lenstra, Vlatka Cubric-Curik

**Affiliations:** ^1^ Department of Animal Science, University of Zagreb Faculty of Agriculture, Zagreb, Croatia; ^2^ Department of Animal Production and Biotechnology, Faculty of Agrobiotechnical Sciences Osijek, Chair for Domestic Animal Breeding and Genetics, J. J. Strossmayer University of Osijek, Osijek, Croatia; ^3^ College of Animal Science and Technology, China Agricultural University, Beijing, China; ^4^ Production Systems, Natural Resources Institute Finland (Luke), Jokioinen, Finland; ^5^ Dipartimento di Scienze Agrarie, Alimentari e Forestali, University of Palermo, Palermo, Italy; ^6^ Dipartimento di Bioscienze, Biotecnologie e Biofarmaceutica, Universita Degli Studi di Bari “Aldo Moro”, Bari, Italy; ^7^ Faculty of Veterinary Medicine, Utrecht University, Utrecht, Netherlands

**Keywords:** Croatian sheep breeds, effective population size, genomic characterization, inbreeding, population structure

## Abstract

A recent comprehensive genomic analysis based on 50K SNP profiles has shown that the regional Balkan sheep populations have considerable genetic overlap but are distinctly different from surrounding breeds. All eight Croatian sheep breeds were represented by a small number of individuals per breed. Here, we genotyped 220 individuals representing the native Croatian sheep breeds (Istrian Sheep, Krk Island Sheep, Cres Island Sheep, Rab Island Sheep, Lika Pramenka, Pag Island Sheep, Dalmatian Pramenka, Dubrovnik Sheep) and mouflon using the Ovine Infinium^®^ HD SNP BeadChip (606,006 SNPs). In addition, we included publicly available Balkan Pramenka and other Mediterranean sheep breeds. Our analyses revealed the complex population structure of Croatian sheep breeds and their origin and geographic barriers (island versus mainland). Migration patterns confirmed the historical establishment of breeds and the pathways of gene flow. Inbreeding coefficients (F_ROH>2 Mb_) between sheep populations ranged from 0.025 to 0.070, with lower inbreeding coefficients observed in Dalmatian Pramenka and Pag Island Sheep and higher inbreeding in Dubrovnik sheep. The estimated effective population size ranged from 61 to 1039 for Krk Island Sheep and Dalmatian Pramenka, respectively. Higher inbreeding levels and lower effective population size indicate the need for improved conservation management to maintain genetic diversity in some breeds. Our results will contribute to breeding and conservation strategies of native Croatian sheep breeds.

## Introduction

Sheep were domesticated about 10,000 years ago in the region of Anatolia and, along with goats, were among the first domesticated farm animals. Sheep were first hunted by humans and over time became managed wild populations, then kept in controlled herds, and finally, humans began to breed sheep (Zeder, 2008; Larson and Burger, 2013). Trought the history breeding for diseriable traits was always present but only about 200 years ago, the organized formation of sheep breeds began, resulting in fragmented populations, and reduced genetic diversity (Taberlet et al., 2008). Genetic diversity is defined as the variety of alleles and genotypes present in a population (Frankham et al., 2002). Each breed has a unique genetic characteristic due to mutations and drift caused by geographic isolation and bottlenecks, artificial selection and adaptation to climate, nutrition and diseases and parasites (Barker, 2001). Many sheep breeds are local breeds that have adapted to specific locations for thousands of years and are closely associated with culture and history (Gandini and Villa, 2003). These small, unique breeds contribute to the overall diversity of the species, but diversity within breeds is low. Highly productive breeds have displaced local breeds and unique gene combinations of some local breeds are being lost (Groeneveld et al., 2010). Erosion of within-breed diversity could be a problem even for breeds whose overall populations remain very large. Monitoring population trends is a prerequisite for rapid and effective action to protect breeds from extinction. Measures to prevent the loss of livestock diversity will be more effective if the factors that drive genetic erosion and extinction risk are well understood. Maintaining genetic diversity is important for rapid adaptation to challenges (Andersson, 2012). Thus, characterizing genetic diversity is an important aspect of developing sustainable strategies for breed improvement (Groeneveld et al., 2010).

Livestock breeding, especially sheep species, was an important part of agriculture in Croatia. The first written records of sheep breeding in Croatia date back to 1781, and two decades later, the number of sheep per citizen in Europe was highest in Dalmatia (Defilipis, 1966), a region of Croatia whose name comes from Illyrian words *dalma,* meaning sheep. Since then, the number of sheep on Croatian territory has steadily decreased. The erosion of agricultural production combined with wars led to a drastic decrease in the number of some native Croatian breeds, which were threatened by extinction. Maintaining genetic diversity in livestock production is critical for meeting future challenges such as climate change, emerging diseases, and food security for a growing human population (Barker, 2001; Groeneveld et al., 2010). Croatia has a number of unique sheep breeds. The native Croatian sheep breeds belong to the Pramenka type, which is characterized by coarse wool, low production, and high resistance to environmental conditions. Within the Pramenka type, there are many breeds/populations that differ in size, wool quality and colour, as well as in their adaptation to the specific microclimatic conditions in their breeding area. Throughout history, Croatian farmers have been eager to improve production characteristics and therefore imported high-performance breeds. Merino rams (Pag Island Sheep, Rab Island Sheep, Krk Island Sheep, Cres Island Sheep and Dubrovnik sheep) and meat breeds such as Merinolandschaf (Dalmatian Pramenka and Lika Pramenka) or Bergamasca Sheep (Istrian Sheep) were mainly used to improve primitive Pramenka type. With the progress of selection, different breeds were created. Genetic diversity in sheep needs to be estimated to identify unique breeds that may be in danger of extinction.

The genetic study of native Croatian breeds started with β-lactoglobulin polymorphisms in Pag Island Sheep (Cubric-Curik et al., 2002). Since then, scientists have studied genetic diversity (Feligini et al., 2005; Ivanković et al., 2005; Cubric-Curik et al., 2009; Kasap et al., 2021; Špehar et al., 2022) and population structure (Lawson Handley et al., 2007; Ćinkulov et al., 2007; Ferencakovic et al., 2012; Salamon et al., 2014) of native Croatian sheep breeds using different approaches. Recently, 50 K SNP markers (Ciani et al., 2020) and whole-genome sequences (Deng et al., 2020; Lv et al., 2021) have been used in population genetic studies presenting native Croatian breeds, but with a very small number of animals per breed. Ciani et al. (2020) showed the separation of Balkan Pramenka from the rest of the European breeds and the belonging of native Croatian sheep breeds to the Pramenka cluster. The authors suggested that Balkan breeds are evolutionary connected with the domestication process and are one of the hub regions from which the migration of sheep spread to the rest of Europe. Recently, Shihabi et al. (2022) identified specific adaptive selection signals on the X chromosome of the individuals used in this analysis.

The main objective of this study was to evaluate the conservation status (diversity, inbreeding, and effective population size), population structure, and admixture of eight native Croatian sheep breeds. Our analysis was based on high-density autosomal SNP chip genotype information sampled from 20 to 45 individuals per breed. Our study is an extension of the study by Ciani et al. (2020), in which only a few individuals representing several native Croatian breeds were analyzed using 50 K SNP chip information. More specifically, the data used refer to a sample of 201 individuals representing eight native Croatian breeds (Cres Island Sheep, Dalmatian Pramenka, Dubrovnik Ruda, Istrian Sheep, Krk Island Sheep, Lika Pramenka, Pag Island Sheep, and Rab Island Sheep) and 10 mouflon sampled in Croatia. They provide an accurate estimate of genetic diversity, ROH-based inbreeding, current and historical effective population size, and allow in-depth comparison with other Mediterranean breeds and some other Pramenka sheep breeds.

## Materials and Methods

### Sample Collection

**FIGURE 1:**
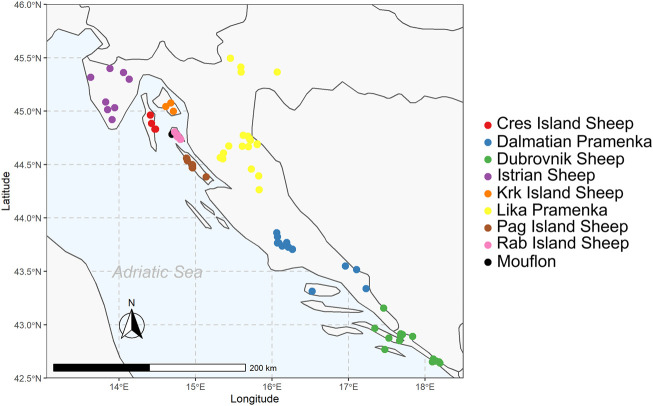
Map of sampling locations for native Croatian sheep breeds. The map illustrates the geographic locations where samples were collected. Each breed is presented with a different colour.

The Croatian sheep represented in this study were: Istrian Sheep (ISS), Pag Island Sheep (PIS), Krk Island Sheep (KIS), Cres Island Sheep (CIS), Rab Island Sheep (RIS), Lika Pramenka (LPS), Dalmatian Pramenka (DPS) and Dubrovnik sheep (DRS) breeds, and Mouflon (EMC). The description of the native Croatian breeds is in Supplementary Data S1. These eight sheep breeds represent different geographical regions in Croatia and can be divided into island breeds (ISS, CIS, KIS, RIS and PIS) and mainland breeds (LPS, DPS, and DRS). Sampling locations are the places of origin of the breeds and are presented in Figure 1. For breeds statistics, see Supplementary Table S1. For this study, blood or tissue samples were randomly collected from 107 female and 105 male sheep located on 105 family farms representing 20 Cres Island Sheep, 20 Krk Island Sheep, 20 Rab Island Sheep, 20 Lika Pramenka, 25 Istrian Sheep, 26 Dubrovnik sheep, 26 Dalmatian Pramenka, 45 Pag Island Sheep, and 10 mouflons. Blood was collected by superficial venipuncture using sterile 10 ml EDTA Vacutainers (BD, Becton, Dickinson and Company, Oxford, United Kingdom) and stored at −86°C until further use. Tissue samples were collected using the Allflex TSU applicator (Allflex, France) and stored at +4°C until further use. All samples were collected according to national and European ethical protocols and directives.

### DNA Extraction and SNP Genotyping

DNA extraction was performed using the Qiagen DNeasy Blood & Tissue Kit (Qiagen, Germany) according to the sample preparation and extraction protocol. DNA quality was checked by 1% agarose gel electrophoresis, while DNA quantity was determined using a NanoPhotometer P330 spectrophotometer (IMPLEN, Germany).

Croatian sheep and mouflon samples were genotyped using the Ovine Infinium HD BeadChip (Illumina, San Diego, CA, United States) by Gene Seek, Neogen Genomics (Neogene Europe Ltd., Scotland, United Kingdom). Genotypes were mapped to the Oar4.0 map. Quality control and filtering of SNPs were obtained using SNP & Variation Suite v8.7.0 (Golden Helix, Inc., Bozeman, MT, www.goldenhelix.com). The accuracy and efficiency of SNP genotyping were assessed by applying a cut-off value of 0.7 for the Illumina GenCall score and 0.4 for the Illumina GenTrain score. All SNPs with more than 10% missing genotypes and individuals with more than 5% missing genotypes were removed. SNPs without position or SNPs that had been assigned to sex chromosomes or mitochondrial genome were also excluded. After quality control, a total of 201 sheep and 10 mouflons remained with 470,962 SNPs.

**FIGURE 2:**
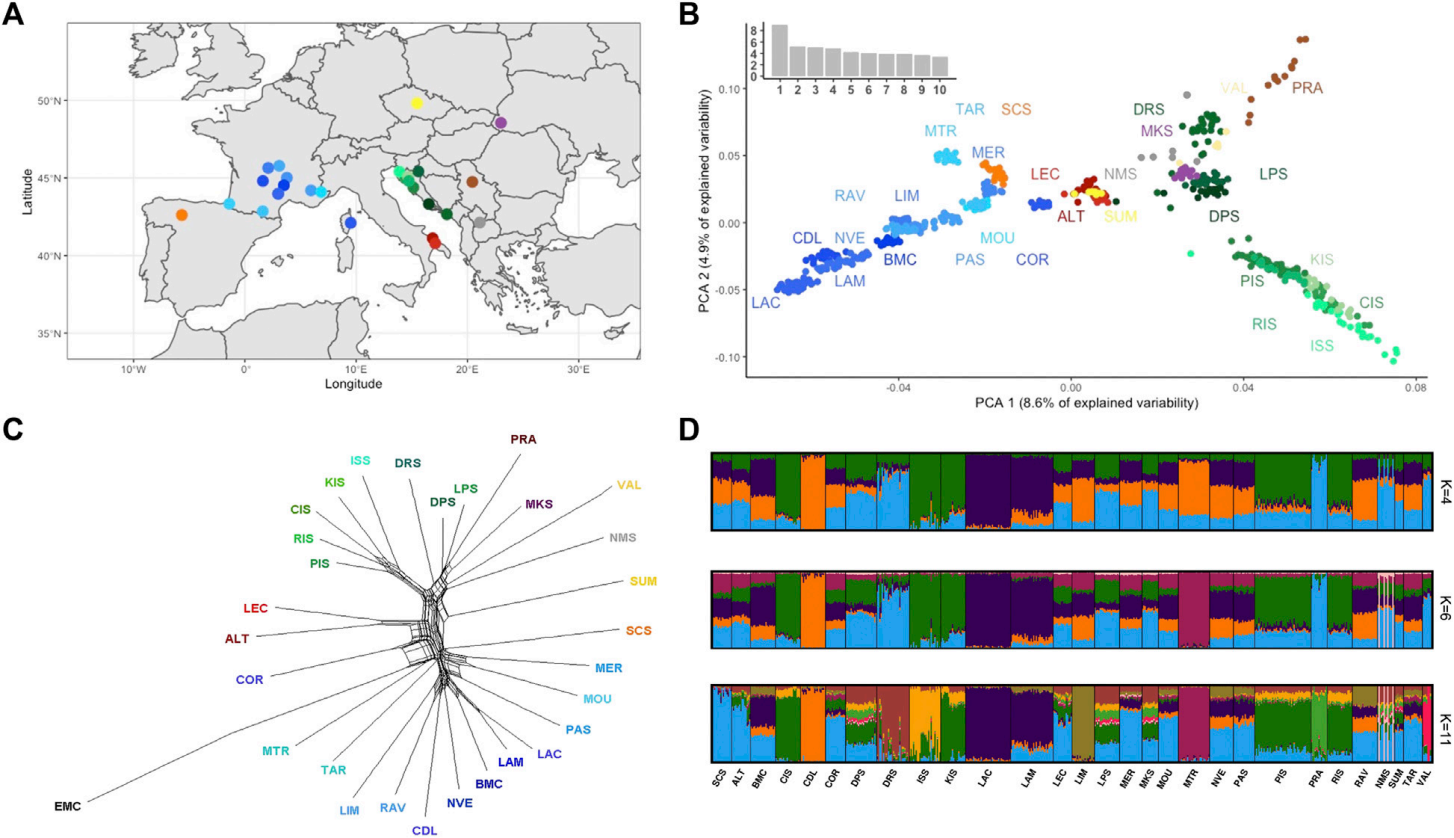
**(A)** Map of the origin of the breeds used in the Mediterranean dataset; **(B)** PCA plot for Mediterranean sheep breeds with principal component 1 and principal component 2.; **(C)** Neighbour network based on the Nei genetic distances constructed for Mediterranean dataset; **(D)** Graphical presentations of the population structure analyses for a sample of 574 Mediterranean sheep. Each sheep is represented by a single vertical line broken into K colour segments, with lengths proportional to the estimated membership of the inferred cluster. Each country is presented with different colour: Croatia—green; France—blue; Spain—orange; Italy—red; North-Macedonia—grey; Ukraine—purple; Czech Republic—yellow; Serbia—brown. Within a country, each breed is represented with a different main colour and three-letter coding: DRS, Dubrovnik Sheep; LPS, Lika Pramenka; DPS, Dalmatian Pramenka; PIS, Pag Island Sheep; RIS, Rab Island Sheep; CIS, Cres Island Sheep; KIS, Krk Island Sheep; ISS, Istrian Sheep; PRA, Serbian Pramenka; VAL, Valachian; MKS, Mount Carpatian Sheep; NMS, North Macedonian Pramenka; SUM, Sumavaska; ALT, Altamunrana; LEC, Leccese; SCS, Churra; COR, Corse; MER, Merino; NVE, Noire du Velay; CDL, Causse du Lot; RAV, Rava; BMC, Blanche du Massif Central; LAM, Meat Lacaune; LAC, Milk Lacaune; LIM, Limousine; TAR, Tarasconnaise; MTR, Manech Tete Rouge; PAS, Preaples du Sud; MOU, Mourerous.

To compare the genetic relationships between Croatian sheep breeds and other Mediterranean sheep and to investigate population structure and admixture patterns, a reference panel of four Balkan Pramenka type sheep (Valachian—VAL, Sumavaska—SUM, Serbian Pramenka—PRA, Carpathian Mountain sheep—MKS) obtained from Cao et al. (2020), one Pramenka from this study (North—Macedonia Pramenka—NMS) and 13 other publicly available Mediterranean sheep breeds (Merino—MER, Noire du Velay—NVE, Causse du Lot—CDL, Rava—RAV, Blanche du Massif central—BMC, Meat Lacune—LAM, Dairy Lacune—LAC, Limousine—LIM, Tarasconnaise—TAR, Corse—COR, Manech Tete Rouge—MTR, Prealpes du Sud—PAS, Mourerous—MOU, Altamurana—ALT, Leccese - LEC) was used. Two datasets were created: I. Croatian dataset with 201 animals and 470962 SNPs and II. Mediterranean dataset with 574 animals (Croatian sheep included) and 348,968 SNPs (after merging and quality control). Map of the breed origin for the Mediterranean dataset is presented in Figure 2A.

### Population Divergence and Relationship

To estimate genetic diversity within the population, expected heterozygosity, observed heterozygosity and the inbreeding coefficient (F_IS_) were calculated using the software PLINK 1.9 (Purcell et al., 2007). In addition, Wright’s F_ST_ fixation indices were calculated to determine the degree of genetic differentiation among selected sheep breeds using SNP & Variation Suite v8.7.0 (Golden Helix, Inc., Bozeman, MT, www.goldenhelix.com) and mean F_ST_ was calculated. Principal Component Analysis (PCA) was performed to investigate the genetic relationship among Croatian sheep breeds as well as at the Mediterranean level. PCA was performed for both datasets: Mediterranean and Croatian, using the R package “SNPRelate” (Zheng et al., 2012). Phylogenetic relationships based on Nei’s genetic distances (Nei, 1978) were presented with a Neighbor network using SplitsTree4 software (Huson and Bryant, 2006). Nei genetic distances were calculated in the program R using the package “stAMPP” (Pembleton et al., 2013).

### Population Structure

Genetic relationships and population structures were assessed with the software STRUCTURE v.2.3.4. (Pritchard et al., 2000) using the Bayesian model based on 83,795 SNPs in eight Croatian sheep breeds and 104,623 SNPs in 28 Mediterranean sheep breeds after pruning based on LD. Pruning of the data was performed in a sliding window of 50 SNPs, moving in increments of five SNPs along each chromosome and removing one SNP pair at a time with a pairwise *r* ^2^ > 0.1 using the software PLINK 1.9 (Purcell et al., 2007). After pruning the SNP missing rate was 0.000–0.003 and 0.000–0.031 per individual for Croatian dataset and Mediterannean dataset, respectively, and 0.003–0.011 for Mourerous and Altamurana population, respectively. For the Croatian and Mediterranean datasets, an ancestry model was constructed for a putative number of ancestral populations of 1–10 and 1–30, respectively. For each K value, 10 runs of 100,000 Markov chain Monte Carlo iterations were performed after a burn-in period of 10,000 iterations. The most likely number of clusters was determined using the ΔK method (Evanno et al., 2005) implemented in software Structure Selector (Li and Liu, 2018). Results from STRUCTURE were analyzed and visualized using the software Structure Selector (Li and Liu, 2018).

### Gene Flow

Gene flow between Croatian and Mediterranean sheep breeds was examined using TreeMix v. 1.13 (Pickrell and Pritchard, 2012). TreeMix was applied to infer population trees and migration events between divergent populations. To determine the position of the root in the maximum likelihood tree, the European mouflon (*n* = 10) was defined as the outgroup population. Between 0 and 15 migration events were inferred.

### Genomic Inbreeding Based on Runs of Homozygosity

The proportion of the genome that is autozygose is estimated by identification of Runs Of Homozygosity (ROH) with SNP & Variation Suite v8.7.0. ROH was detected for each individual separately using the following criteria: (I) the minimum number of SNPs in ROH was set to 50, which was calculated using the method proposed by Lencz et al. (2007) to minimize the number of false-positive ROH:

$$l=\;\frac{\ln\frac{\alpha }{n_{s}*n_{i}}}{\ln\left(1-het\right)}$$

where *α* is the percentage of false-positive ROH (set to 0.05 in this study), n_s_ is the number of SNPs per individual, n_i_ is the number of individuals, *het* is heterozygosity across all SNPs; (II) the maximal gap between adjacent SNPs was set to 250 Kb; (III) the minimum SNP density per ROH was set to 1 SNP every 50 Kb; (IV) the minimum length constituting ROH was set to 2 Mb. To account for genotyping error, ROHs were calculated separately for each of the five categories specified according to the ROH length: >2 Mb, 2–4 Mb, 4–8 Mb, 8–16 Mb, and >16 Mb. Based on ROH length, the number of allowed heterozygotes and missing genotypes were defined according to Ferenčaković et al. (2013).

The individual genomic inbreeding coefficients based on ROH (F_ROH_) were estimated as the sum of the length of all ROH per individual divided by the total length of the autosomal genome covered by SNPs, as described by McQuillan et al. (2008). The F_ROH_ was calculated for different minimum ROH lengths because the short segments are associated with individual autozygosity derived from a distant common ancestor, whereas long ROH segments are correlated with recent inbreeding (Ferenčaković et al., 2013). For each animal, we calculated the F_ROH2–4 Mb_, F_ROH4–8 Mb_, F_ROH8–16 Mb_, and F_ROH>16 Mb_ based on ROH with different minimum lengths. Based on the estimated individual F_ROH>2 Mb_, the breed inbreeding coefficient was calculated by averaging the F_ROH_ estimates per breed.

### Effective Population Size

To better understand the demographic history of native Croatian sheep breeds, we estimated current and historical effective population size (N_e_) form linkage disequilibrium (LD). Trends in effective population size were estimated using an optimization method that implements a genetic algorithm (Mitchell, 1998) to infer the recent demographic history of a population from SNP data of a small sample of contemporary individuals, as implemented in the software GONE (Santiago et al., 2020). Default parameters were applied. The most recent estimate of N_e_ was taken in the current generation. Furthermore, N_e_ estimates from 50 generations ago were used for comparison with results from other authors (Kijas et al., 2012; Beynon et al., 2015; Deniskova et al., 2019).

## Results and Discussion

### Population Divergence and Relationships

Genetic diversity parameters are presented in Table 1. Observed heterozygosity ranged from 0.327 ± 0.006 in Rab Island Sheep to 0.346 ± 0.003 in Dalmatian Pramenka. The average expected heterozygosity within the breeds ranged from 0.336 in Istrian Sheep to 0.348 in Dalmatian Pramenka. The inbreeding coefficient (F_IS_) varied from −0.017 ± 0.011 in Istrian Sheep to 0.022 ± 0.018 in Rab Island Sheep. The results of observed and expected heterozygosity are similar to those found by Al-Mamun et al. (2015) in five Australian sheep breeds using a 50k SNP chip and Edea et al. (2017) in Ethiopian sheep using a 50 and 600K SNP chip. Similar values of expected heterozygosity were reported for native Italian breeds (Ciani et al., 2014), southern and western European sheep breeds (Kijas et al., 2012) and native Russian sheep breeds (Deniskova et al., 2018).

**TABLE 1:** Genetic diversity indices for native Croatian sheep breeds. H_o_, observed heterozygosity; H_e_, expected heterozygosity; F_IS_, inbreeding coefficient; F_ROH>2 Mb_, genomic inbreeding calculated for ROH length over 2 Mb; N_e0_, current effective population size. Each breed is represented with a three-letter coding: DRS, Dubrovnik Sheep; LPS, Lika Pramenka; DPS, Dalmatian Pramenka; PIS, Pag Island Sheep; RIS, Rab Island Sheep; CIS, Cres Island Sheep; KIS, Krk Island Sheep; ISS, Istrian Sheep.

Breed	H_o_ ± SE	H_e_ ± SE	F_IS_ ± SE	F_ROH>2 Mb_ ± SE	N_e0_
CIS	0.340 ± 0.004	0.341 ± 0.000	0.002 ± 0.011	0.049 ± 0.011	148
DPS	0.346 ± 0.003	0.348 ± 0.000	0.004 ± 0.008	0.025 ± 0.008	1039
DRS	0.339 ± 0.005	0.341 ± 0.000	0.005 ± 0.015	0.070 ± 0.015	157
ISS	0.342 ± 0.004	0.336 ± 0.000	-0.017 ± 0.011	0.053 ± 0.010	161
KIS	0.340 ± 0.004	0.340 ± 0.000	0.001 ± 0.012	0.058 ± 0.011	62
LPS	0.345 ± 0.004	0.347 ± 0.000	0.005 ± 0.012	0.033 ± 0.012	598
PIS	0.339 ± 0.003	0.345 ± 0.000	0.016 ± 0.008	0.035 ± 0.008	1005
RIS	0.337 ± 0.006	0.345 ± 0.000	0.022 ± 0.018	0.055 ± 0.017	559

Genetic relationships between individuals of multiple sheep breeds were assessed by principal component analysis (PCA). The PCA plot of the first and second principal components (PC) is shown in Figure 2B. The breeds were generally grouped according to their geographic origin. Similar results were also presented in Rochus et al. (2020) and Ciani et al. (2020). Total variation explained by the PC components was 94.52%. The largest component (8.6% of the total variation) showed an east-west cline between the Western European sheep and the Balkan Pramenka sheep. The second PC separated Croatian island breeds (ISS, CIS, RIS, KIS, PIS) from the rest of the Pramenka cluster and highlighted a division of native Croatian breeds into mainland breeds (DRS, LPS, and DPS) and island breeds. Two Croatian breed groups have contrasting structures. In the mainland group, breeds were clearly separated and individuals had clear cluster assignments, indicating greater differentiation between breeds. In contrast, the island breeds showed less differentiation with tighter clusters and breed overlapping. A similar differentiation in the structure of the two French sheep groups was observed in Rochus et al. (2018). Two Italian breeds occupy a central position between Balkan Pramenka sheep and French and Spanish sheep. The genetic proximity of Pramenka sheep, Italian and Spanish breeds is interpreted as a possible sheep migration route along the Mediterranean coast from the Balkans to Spain *via* Italy, as presented in Ciani et al. (2020).

To better estimate the relationships between native Croatian sheep breeds PCA was performed only on the Croatian dataset (Supplementary Figure S1). The first two components together explained 14.9% of the total variation, representing a large portion of the variability. Principal component analysis showed a clear separation of Istrian Sheep and Dubrovnik Sheep, while Lika Pramenka and Dalmatian Pramenka created one close cluster and Rab Island Sheep, Pag Island Sheep, Krk Island Sheep, and Cres Island Sheep formed a second cluster. Mainland and island cluster are not well defined here. The result shows a north-south cline following the geographical breeding area of each breed. A similar pattern following the geographical distribution of breeds in the PCA graph was found in Ciani et al. (2014) and Edea et al. (2017). The largest PC (8.1% of the total variation) positioned Dubrovnik Sheep apart from the rest of the Croatian native sheep breeds. The second PC (6.8% of the total variation) separated the Istrian Sheep from all others. The genetic uniqueness of Istrian Sheep was observed earlier in Ćinkulov et al. (2007), Lawson Handley et al. (2007), and Salamon et al. (2014) using microsatellite markers. Istrian Sheep and Dubrovnik Sheep are also geographically isolated from other native Croatian sheep breeds, but they are also phenotypically the most diverse.

The relationship between the Mediterranean sheep breeds and the mouflon was determined using genetic distances according to Nei (1972), which include a correction for drift and mutations. Higher genetic distances also indicate a longer temporal divergence of the breeds. The European mouflon was set as an outgroup. Neighbor-net analysis of Mediterranean sheep (Figure 2C) confirms the separation of the Pramenka from the rest of the European sheep population. The genetic uniqueness of the Croatian island breeds is emphasized here by their separation from the Pramenka group. Analysis by Ciani et al. (2020) using a 50K SNP chip shows similar separation of Croatian island sheep breeds from other European breeds and their positioning between Pramenka and Italian sheep. The Lika and Dalmatian Pramenka were in a group with the Serbian Pramenka and the Ukrainian Mountain Carpathian sheep, confirming the relationships identified by Ciani et al. (2020). The separation of Croatian island and continental breeds suggests that geographic barriers have an influence on shaping the current structure of native Croatian sheep breeds. Greater geographic distances with barriers such as the sea and mountains have reduced the exchange of genetic material between these two groups of breeds, resulting in greater separation of these breeds. Earlier gene flows and population mixing, which most likely occurred between the Balkan Pramenka populations, are still visible in the genetic structure of the Croatian mainland breeds and should not be neglected here. In the past, a Pramenka was bred on the territory of today’s Republic of Croatia, the so-called Balkan Pramenka, which was divided into different types depending on where it was bred (e.g., Travnik type, Sjenica type …). Later, with the disintegration of Yugoslavia, the gene flow between Pramenka types decreased and new breeds were formed, which still show a great genetic connection. The more frequent and stronger introduction of Italian breeds into island populations, especially Pag Island Sheep, is reflected in the proximity of these two sheep groups in the graph, although in this dataset were no representatives of Italian Merino sheep involved in breeding native Croatian sheep breeds.

The level of genetic differentiation based on F_ST_ estimation between breeds were calculated. Genetically similar populations will have lower F_ST_ values, while breeds that are more genetically diverse should have higher F_ST_ values (Berendse et al., 2009). F_ST_ values for Croatian breeds and selected breed pairs are presented in Table 2. The breeds that were marginal on the PCA plot were selected. The lowest F_ST_ value was observed between Lika Pramenka and Dalmatian Pramenka (F_ST_ = 0.003), indicating high genetic similarity between the two breeds. The most divergent breeds among the selected populations were Istrian Sheep and North-Macedonian Pramenka (F_ST_ = 0.081). The significant genetic distance of Istrian Sheep and Dubrovnik Sheep observed in PCA was consistent with the higher F_ST_ values obtained when comparing Istrian Sheep and Dubrovnik Sheep with native Croatian sheep breed. The mean F_ST_ value was calculated for each selected breed. The lowest mean F_ST_ was observed for Dalmatian Pramenka (MF_ST_ = 0.029). The breed with the lowest mean F_ST_ is considered as the central breed, since it has the highest genetic similarity with all other breeds.

**TABLE 2:** Genetic differentiation (F_ST)_ between native Croatian sheep breeds and selected European breeds. MF_ST_—average F_ST_ estimates among breeds.

Breed	DPS	TAR	NMS	RIS	PIS	NVE	MER	LPS	LEC	LAC	KIS	ISS	DRS	CIS	MF_ST_
DPS-Dalmatian Pramenka															0.029
TAR-Tarasconnaise	0.033														0.048
NMS-North Macedonian Sheep	0.042	0.067													0.064
RIS-Rab Island Sheep	0.016	0.041	0.056												0.034
PIS-Pag Island Sheep	0.015	0.039	0.053	0.011											0.033
NVE-Noire du Velay	0.037	0.044	0.072	0.045	0.043										0.052
MER-Merino	0.033	0.040	0.062	0.042	0.039	0.046									0.048
LPS-Lika Pramenka	0.003	0.036	0.045	0.021	0.020	0.041	0.037								0.032
LEC-Leccese	0.023	0.039	0.060	0.032	0.030	0.045	0.040	0.027							0.041
LAC-Lacaune (Dairy)	0.041	0.047	0.074	0.049	0.048	0.047	0.049	0.045	0.048						0.055
KIS-krk Island Sheep	0.029	0.055	0.070	0.024	0.025	0.059	0.056	0.033	0.046	0.063					0.047
ISS-Istrian Sheep	0.040	0.066	0.081	0.040	0.041	0.070	0.068	0.044	0.057	0.074	0.051				0.058
DRS-Dubrovnik Sheep	0.026	0.053	0.064	0.041	0.039	0.058	0.051	0.030	0.045	0.061	0.053	0.065			0.050
CIS-Cres Island Sheep	0.031	0.056	0.072	0.020	0.022	0.060	0.058	0.036	0.046	0.064	0.034	0.053	0.055		0.047
ALT-Altamurana	0.034	0.051	0.070	0.043	0.041	0.055	0.050	0.038	0.030	0.058	0.056	0.068	0.055	0.057	0.050

### Population Structure

The population structure of Mediterranean breeds was identified using a model-based STRUCTURE analysis with an assumed number of populations set to K = 30. The Evanno ΔK method did not reveal a clear number of genetic clusters, peaking at K = 4, K = 6, and K = 11 (Supplementary Figure S2), indicating a hierarchical population structure. The results of model-based population structuring revealed a complex genetic structure of native Croatian sheep breeds when placed in the Mediterranean context (Figure 2D). At K = 2, we observed the separation of Balkan Pramenka from Western European breeds. A similar split is presented in the genetic diversity results of 57 European and Middle Eastern sheep breeds by Peter et al. (2007). At K = 3, the French breeds Causse du Lot (CDL) and Manech Tête Rouge (MTR) showed different genetic backgrounds. The separation of breeds continued as the number of ancestral populations increased, with the separation of Dubrovnik Sheep at K = 9 (data not shown) and Istrian Sheep at K = 11. Dalmatian Pramenka and Lika Pramenka have similar population structure as North Macedonian Pramenka (NMS), Sumavska sheep (SUM) and Mountain Carpathian sheep (MKS). They show a very complex ancestral structure. Very similar results were obtained by Ciani et al. (2020) FineStructure analysis of Eastern and Southeastern European sheep breeds on a 50K SNP chip, showing incomplete differentiation of the Balkan Pramenka sheep and a common ancestral genome. This result is an indication that the defined breeds do not match the actual population boundaries and suggests an exchange of genetic material between these populations. Considering the political situation in which Bosnia and Herzegovina, Croatia, Kosovo, Montenegro, North Macedonia, Serbia and Slovenia were united until 25 years ago, the exchange of genetic material between Pramenka sheep was very likely, and since there is no organized selection in the populations of Dalmatian Pramenka and Lika Pramenka, five generations are not sufficient for genetic differentiation of these breeds.

PCA and population structure analysis have clearly shown that among the native Croatian sheep breeds there is a certain degree of population mixing, which is lowest in Istrian Sheep and Dubrovnik Sheep.

To identify population structure within native Croatian sheep breeds on a finer scale, we performed another STRUCTURE analysis only on the Croatian dataset with an assumed number of populations (K) between one and ten. The most informative number of ancestral populations (K = 3) was estimated using the Evanno ΔK method (Supplementary Figure S3). The results for K = 2–6 are presented in Supplementary Figure S4. The clearest ancestry components when assuming only two ancestral populations showed a clear separation of the Dubrovnik Sheep. At K = 4, the Istrian Sheep are completely separated. The specific genetic structure of the Istrian Sheep was also shown in the research of Ciani et al. (2014), where it stands out as a distinct breed at a very low K = 8. At K = 5, Dalmatian Pramenka and Lika Pramenka form a clearly separate group. These two breeds are not separated even at K = 10, the maximum number of hypothesized groups for this data set that suggest common ancestry. At K = 6, the Krk Island Sheep is divided into three subpopulations, with two subpopulations showing the dominance of a single ancestral genome, while the third population is genetically similar to other island breeds. The cause of such separation within the Krk Island Sheep breed could be due to reduced exchange of genetic material within Krk Island Sheep populations, when under the influence of inbreeding and genetic drift, gene fixation occurs and population structure changes.

### Gene Flow

**FIGURE 3:**
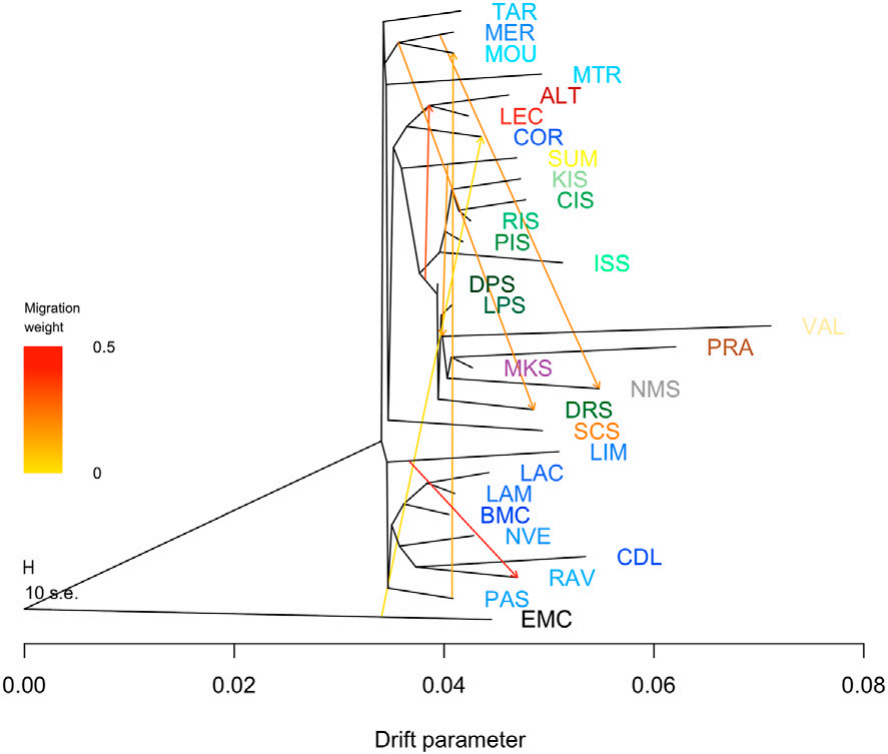
Inferred maximum likelihood population graph considering 12 migration events. Migration arrows are coloured according to their weight. The length of the horizontal branches is proportional to the amount of genetic drift that occurred on each branch. The tree was derived using TreeMix v.1.12. The European mouflon was set as the outgroup. Each country is presented with different colour: Croatia—green; France—blue; Spain—orange; Italy—red; North-Macedonia—grey; Ukraine—purple; Czech Republic—yellow; Serbia—brown. Within a country, each breed is represented with a different main colour and three-letter coding: DRS, Dubrovnik Sheep; LPS, Lika Pramenka; DPS, Dalmatian Pramenka; PIS, Pag Island Sheep; RIS, Rab Island Sheep; CIS, Cres Island Sheep; KIS, Krk Island Sheep; ISS, Istrian Sheep; PRA, Serbian Pramenka; VAL, Valachian; MKS, Mount Carpatian Sheep; NMS, North Macedonian Pramenka; SUM, Sumavaska; ALT, Altamunrana; LEC, Leccese; SCS, Churra; COR, Corse; MER, Merino; NVE, Noire du Velay; CDL, Causse du Lot; RAV, Rava; BMC, Blanche du Massif Central; LAM, Meat Lacaune; LAC, Milk Lacaune; LIM, Limousine; TAR, Tarasconnaise; MTR, Manech Tete Rouge; PAS, Preaples du Sud; MOU, Mourerous.

Migration patterns between native Croatian and Mediterranean sheep breeds were studied using population tree models accounting for migration events and implemented in the program TreeMix. The maximum likelihood tree, created without assuming migration events, was calculated and rooted in the European Mouflon (EMC) (Supplementary Figure S5). The ML tree generally confirmed the relationships revealed by the PCA and STRUCTURE analysis, as in the Mediterranean context the Croatian native sheep breeds were clustered with the other Balkan Pramenka sheep. The Croatian island breeds additionally separated from the other Balkan Pramenka breeds, creating a new branch. As in the PCA graph, the Italian breeds together with the Corse sheep represent the connection between the Balkan and West-European breeds. The West-European sheep breeds show a similar phylogenetic structure as shown in Rochus et al. (2018). In the population tree, the sheep breeds, including the native Croatian breeds, generally had short branch lengths, indicating higher genetic diversity within the breed. By adding migration events, the population tree showed a more detailed view of the genetic relationships between the Mediterranean and Croatian breeds. Estimation of the optimal number of migration events using the ΔM method showed that the population tree with m = 7 migrations best explained the genetic relationships between the breeds (Figure 3). Inference of population trees with m = 7 showed that the migration event with the greatest weight was from the root of the Balkan Pramenka sheep breeds to the root of the two Italian sheep breeds. There are no historical records of the importation of Balkan sheep into Italy, so it is assumed that this is an ancient migration, and it would be interesting to estimate how old it is. The migration edge from the French Merino branch to the Dubrovnik Sheep branch confirms the evolutionary history of the Dubrovnik Sheep, which has a higher presence of the Merino genotype than other native Croatian sheep breeds. The Dubrovnik Sheep is also known by the name Ruda, which was given to the Dubrovnik Sheep in reference to its finer wool. The migration edge with low weight from European mouflon to Corse sheep is presented. This migration is explained by the knowledge that European mouflon living in European countries are imported from populations in Sardinia and Corsica (Guerrini et al., 2015) and that the introduction of mouflon into domestic sheep populations has been recorded in Sardinia and Corsica (Barbato et al., 2017). Migrations between French breeds are the result of breed formation or crossbreeding and are discussed in detail in Rochus et al. (2018).

### Genomic Inbreeding

In sheep populations, estimation of inbreeding coefficient based on pedigree information has not been accurate due to incomplete pedigrees, low generations and frequent errors in recording (Ferenčaković et al., 2013), so estimation of inbreeding coefficients based on high-density SNP markers by ROHs gives us more accurate results.

**FIGURE 4:**
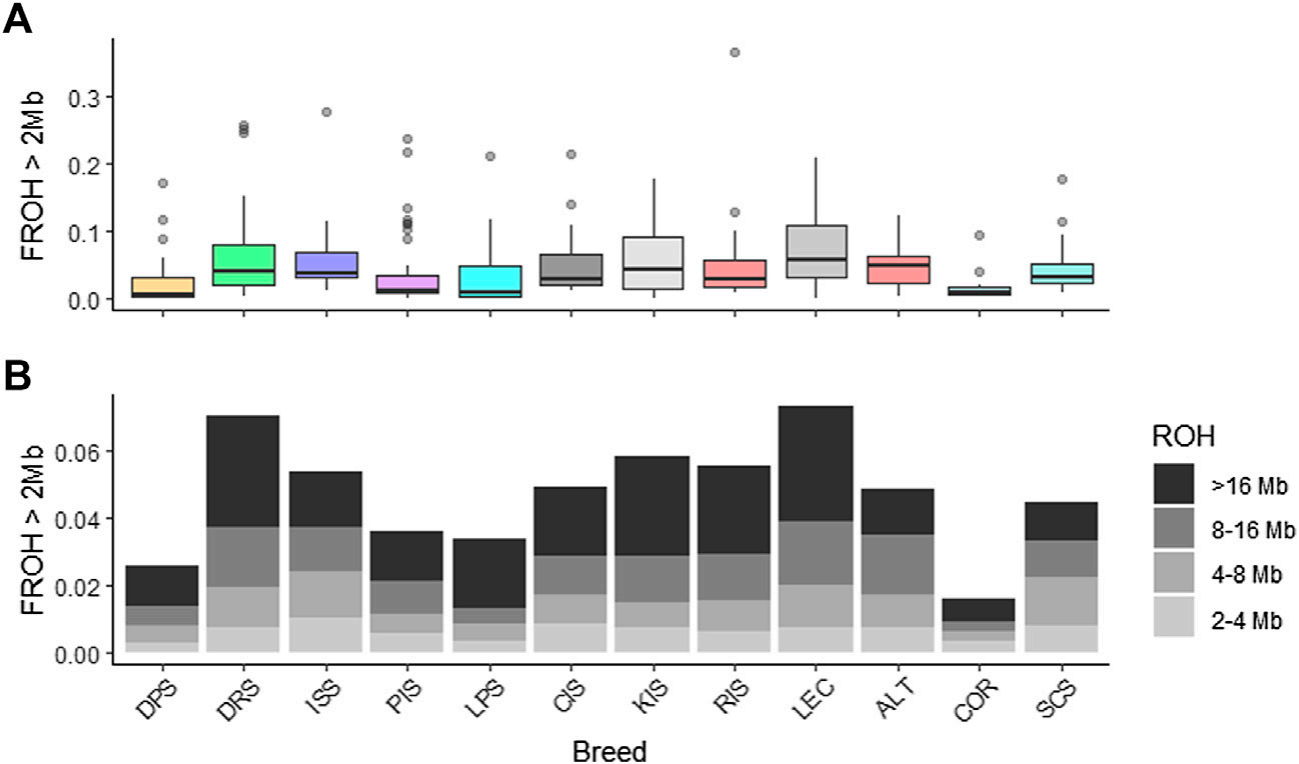
Levels of genomic inbreeding. The genomic inbreeding coefficient (F_ROH_) was estimated as the proportion of runs of homozygosity (ROH) in the total length of autosomes. **(A)** Individual F_ROH_ are averaged per breed. **(B)** The population mean F_ROH_ is presented for each ROH length category. Breeds are represented with a three letter coding: DRS, Dubrovnik Sheep; LPS, Lika pramenka; DPS, Dalmatian pramenka; PIS, Pag Island Sheep; RIS, Rab Island Sheep; CIS, Cres Island Sheep; KIS, Krk Island Sheep; ISS, Istrian Sheep; ALT, Altamunrana; LEC, Leccese; SCS, Churra; COR, Corse.

The overall genomic inbreeding (F_ROH>2 Mb_) for Croatian native sheep breeds and three selected breeds from the Mediterranean dataset (Altamurana, Leccese, Churra and Corse sheep) was estimated as well as inbreeding for each of the defined categories (F_ROH2–4 Mb_, F_ROH4–8 Mb_, F_ROH8–16 Mb_, F_ROH>16 Mb_, Figure 4).

Inbreeding coefficients (F_ROH>2 Mb_) between sheep populations ranged from 0.025 to 0.070 (Table 1), with lower inbreeding coefficients observed in Dalmatian Pramenka and Pag Island Sheep. Similar inbreeding coefficients were reported for native Russian breeds (Deniskova et al., 2019) and most Italian sheep breeds (Mastrangelo et al., 2018), while higher levels of inbreeding were observed in Barbaresca, Delle Langhe, Valle del Belice (Mastrangelo et al., 2017), Leccese (Mastrangelo et al., 2018, this study) and African sheep breeds (Dzomba et al., 2021). The low F_ROH_ values observed for almost all native Croatian sheep breeds suggest that the animals in this study are not highly related. During recombination, long ROH segments are interrupted. Long ROH segments are associated with recent inbreeding, while short ROH segments indicate ancient inbreeding (Ferenčaković et al., 2013; Curik et al., 2014). The largest inbreeding values for F_ROH2–4 Mb_ = 0.010 and F_ROH4–8 Mb_ = 0.013 were observed in Istrian Sheep, indicating the presence of IBD individuals and more ancient relatedness, as suggested by Howrigan et al. (2011). In contrast, the highest inbreeding values for F_ROH8–16 Mb_ = 0.018 and F_ROH>16 Mb_ = 0.033 were observed in Dubrovnik Sheep, indicating recent inbreeding. The Dubrovnik Sheep experienced a drastic decline in the number of individuals in the 1990s. The revival of the breed, which began in 2003, included 112 individuals (Mioč et al., 2003), which represent the genetic base of the current Dubrovnik Sheep population. The highest degree of inbreeding in Dubrovnik Sheep (F_ROH_ = 0.070) is a consequence of the low number of animals available for revitalization. The results show an increase in the number of homozygous regions from the 20th generation to the third generation in all breeds. Accordingly, there is an increase in the average inbreeding coefficient from 0.005 to 0.021, respectively from the 25th to the third generation. The increase in the recent inbreeding coefficient could be due to the extensive use of few rams within herds, as suggested by Mastrangelo et al. (2017). In Croatia, as in Sicily, livestock breeders use natural mating where rams are used in herds with closely related individuals for several years or one ram is used in several herds, what leads to increased inbreeding and consequently lower variability.

### Effective Population Size

Effective population size (N_e_) is an important parameter used in population genetics. It is useful for monitoring genetic diversity and explaining population trends. In addition, effective population size is an indicator of the risk of genetic erosion (Tenesa et al., 2007). It has been known for many years that monitoring effective population size is an important tool for the long-term conservation of endangered populations (Notter, 1999; Gandini et al., 2004; Biscarini et al., 2015).

**FIGURE 5:**
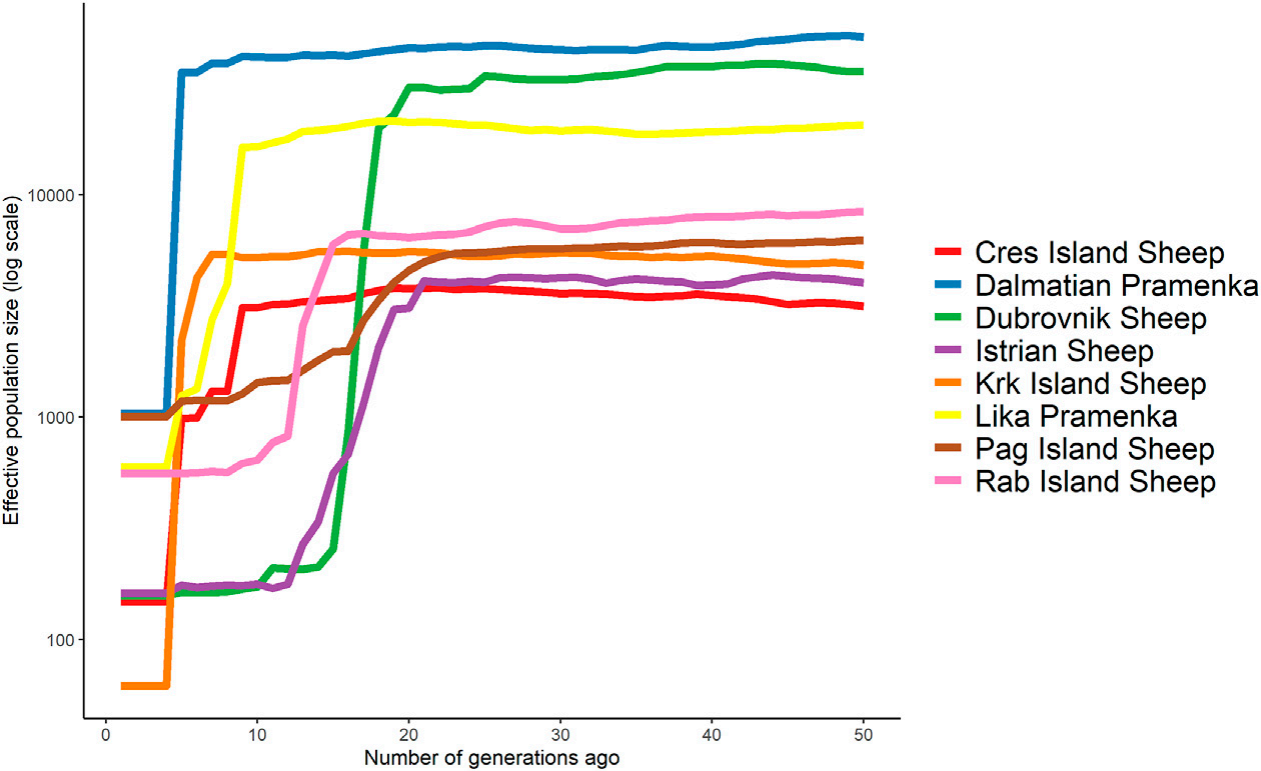
Historical effective population size calculated with the software GONE for native Croatian sheep breeds during 50 generations.

The current effective population size for Croatian sheep breeds estimated using the software GONE ranged from 61 to 1039 for Krk Island Sheep and Dalmatian Pramenka, respectively (Table 1). Higher N_e_ values were also observed for Pag Island Sheep (1005), Lika pramenka (598) and Rab Island Sheep (558). These four breeds (DPS, PIS, LPS, and RIS) show similar N_e_ values as the two Russian Gissar and Aykol (Deniskova et al., 2019). The very low effective population size identified for the Dubrovnik Sheep is similar to that observed for the Russian Kuchugur breed (N_e_ = 65) and the Swedish Gute sheep (N_e_ = 68) and is most likely the result of the Homeland War when the Dubrovnik Sheep population was drastically reduced. The software GONE identified several sudden changes in historical effective population size (Figure 5). The Dalmatian Pramenka and Lika Pramenka, as well as the Dubrovnik Sheep, reached a high historical effective population size and experienced a sharp decline about 25 generations ago. The lowest current effective population size was observed in Krk Island Sheep (N_e_ = 61).

When considering historical effective population size, the highest N_e_ across all generations was observed in Dalmatian Pramenka. Fifty generations ago, the lowest N_e_ values were 3159 (Cres Island Sheep), 4030 (Istrian Sheep) and 4814 (Krk Island Sheep) and the highest were 51537 (Dalmatian Pramenka) and 35988 (Dubrovnik Sheep). These values are higher than those determined by Kijas et al. (2012) and Ciani et al. (2014) for the European sheep breeds and Deniskova et al. (2018) for the Russian breeds. Very high values of historical effective population size for certain breeds indicate that these breeds were very widespread on the territory of Croatia in the past and confirm the historical records of a large number of sheep (1,105,078 head) bred in the area of present Dalmatia (Defilipis, 1966). Also, a very high linear trend in the effective population size of Pag Island Sheep can be attributed to the systematic activities of breeders since 1870, when the Gregge Modella Society was established for the improvement of sheep breeding (Pavlinić, 1936). The much higher values obtained for the Croatian sheep breeds compared to other European breeds could be due to the application of different software. The presence of population substructuring into herds could also affect the estimate of genomic effective population size. In the simulations, the software GONE showed an accurate estimation of effective population size up to 200 generations. Unlike other programs that are based on LD for N_e_ estimation, GONE very accurately reflects changes in N_e_ over history, even when there were substantial increases or decreases in value (Wang et al., 2016; Santiago et al., 2020). All N_e_ estimates based on LD are sensitive to population mixing because the presence of mixing in populations increases N_e_ values. In addition, migration affects N_e_ estimates, as the exchange of rams between flocks and the natural mating systems in sheep breeding can lead to an overestimation of N_e_ values.

## Conclusion

The present study is the first detailed analysis of genomic diversity and population structure of eight native Croatian sheep breeds using high-density SNP markers. PCA, Neighbour network, Maximum likelihood trees and STRUCTURE analyses consistently revealed a common clustering pattern showing a clear separation between the island and mainland breeds. The eight Croatian breeds were generally unique. Some degree of admixture was observed in the population structure, confirming common ancestry. For all Croatian breeds except Krk Island Sheep, the estimated effective population size was above 100 - a threshold above which the breed is sustainable. Inbreeding coefficients estimated from runs of homozygosity were low to moderate. We compared the native Croatian breeds with the other Mediterranean and Pramenka breeds genotyped with high-density markers. In the global context, Croatian sheep clustered with other Pramenka type breeds and differed from West-European breeds. We observed gene flow from the mouflon population to domestic sheep and from Croatian native sheep breeds to Italian breeds. This migration is very interesting and it would be useful to determine the age of the migration as it could be confirmations of the Mediterranean migration route from the domestication center. This study contributes to a better understanding of the genetic background of Croatian native sheep breeds and provides information to support the genomic improvement of these local breeds.

## Data Availability

Genotypic data of 201 animals representing eight Croatian sheep breeds are deposited and available at https://doi.org/10.5061/dryad.pg4f4qrsn.
