# Supplementary material for: High-Density Genomic Characterization of Native Croatian Sheep Breeds
Source: Front Genet. 2022 Jul 15;13:940736. doi: 10.3389/fgene.2022.940736 (PMC9337876; doi:10.3389/fgene.2022.940736)
Supplement: Supplementary file 1 [file Presentation1.zip › Supplementary Figure 1.docx]

Supplementary Material


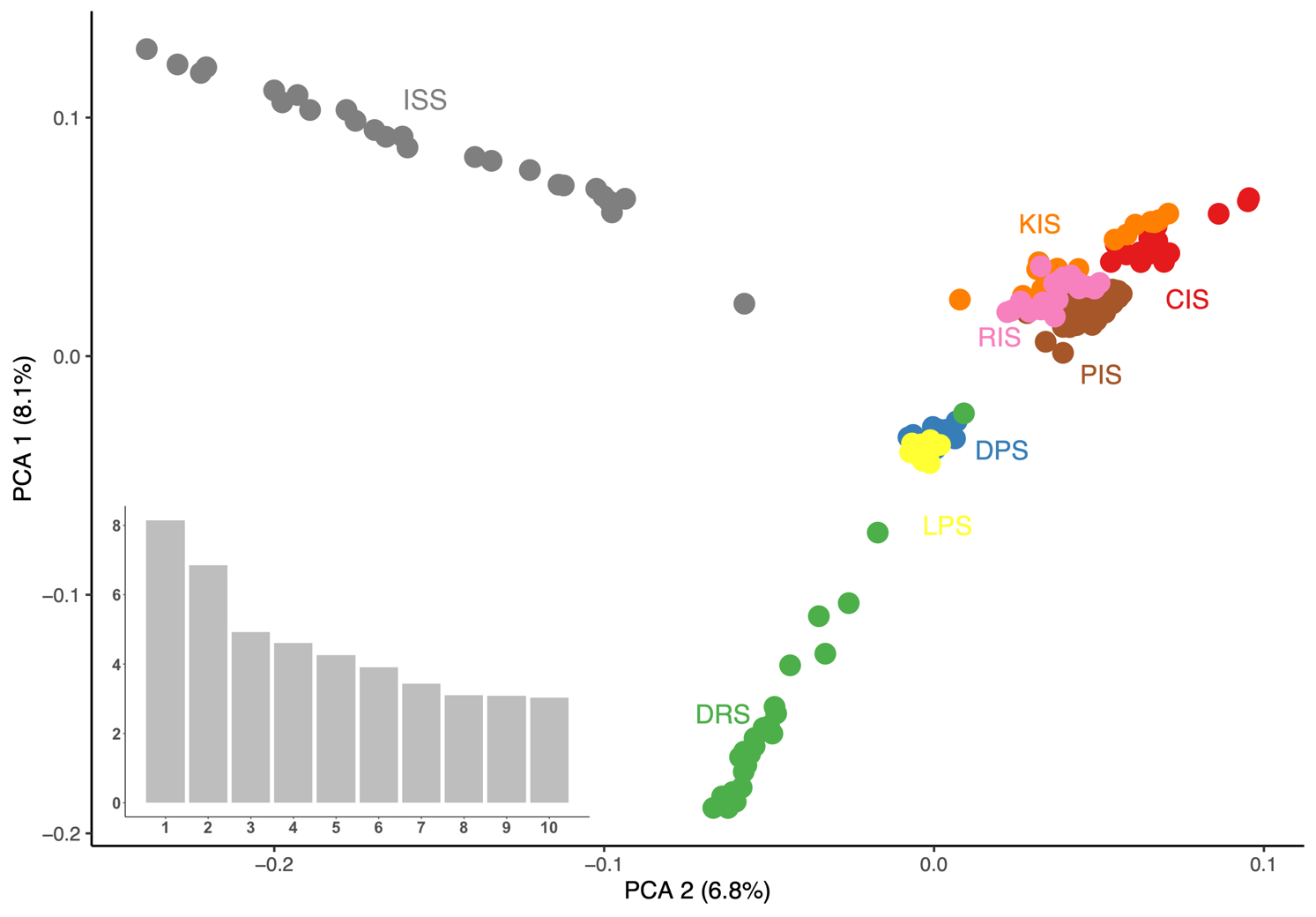


**Supplementary Figure 1.** Principal component analysis for native Croatian sheep breeds showing principal component 1 and principal component 2. Each breed is presented with a different colour and breed abbreviation: DRS – Dubrovnik Sheep, LPS – Lika Pramenka, DPS – Dalmatian Pramenka, PIS – Pag Island Sheep, RIS – Rab Island Sheep, CIS – Cres Island Sheep, KIS – Krk Island Sheep, ISS – Istrian Sheep.
