# Supplementary material for: High-Density Genomic Characterization of Native Croatian Sheep Breeds
Source: Front Genet. 2022 Jul 15;13:940736. doi: 10.3389/fgene.2022.940736 (PMC9337876; doi:10.3389/fgene.2022.940736)
Supplement: Supplementary file 1 [file Presentation1.zip › Supplementary Figure 3.docx]

Supplementary Material


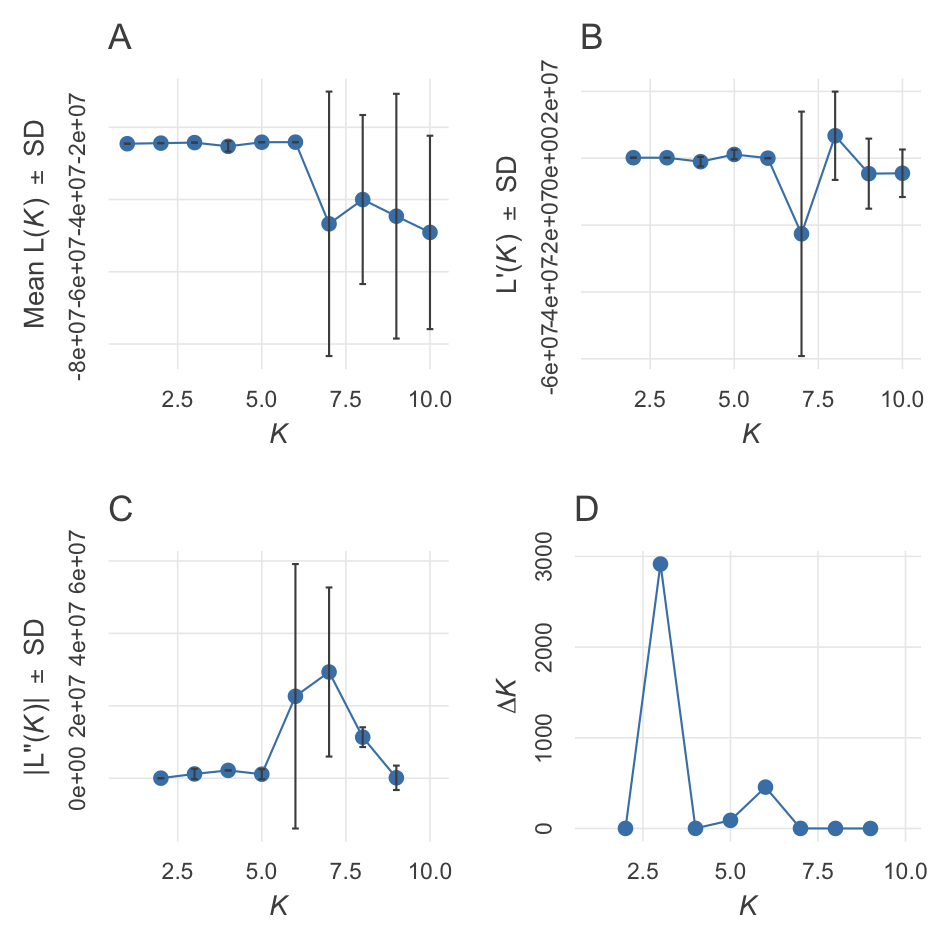


**Supplementary Figure 3.** Plots generated in Structure Selector that show the mean log likelihood of the data [L(K)] and Evanno’s delta K statistic for Croatian dataset
