# Supplementary material for: High-Density Genomic Characterization of Native Croatian Sheep Breeds
Source: Front Genet. 2022 Jul 15;13:940736. doi: 10.3389/fgene.2022.940736 (PMC9337876; doi:10.3389/fgene.2022.940736)
Supplement: Supplementary file 1 [file Presentation1.zip › Supplementary Figure 4.docx]

Supplementary Material


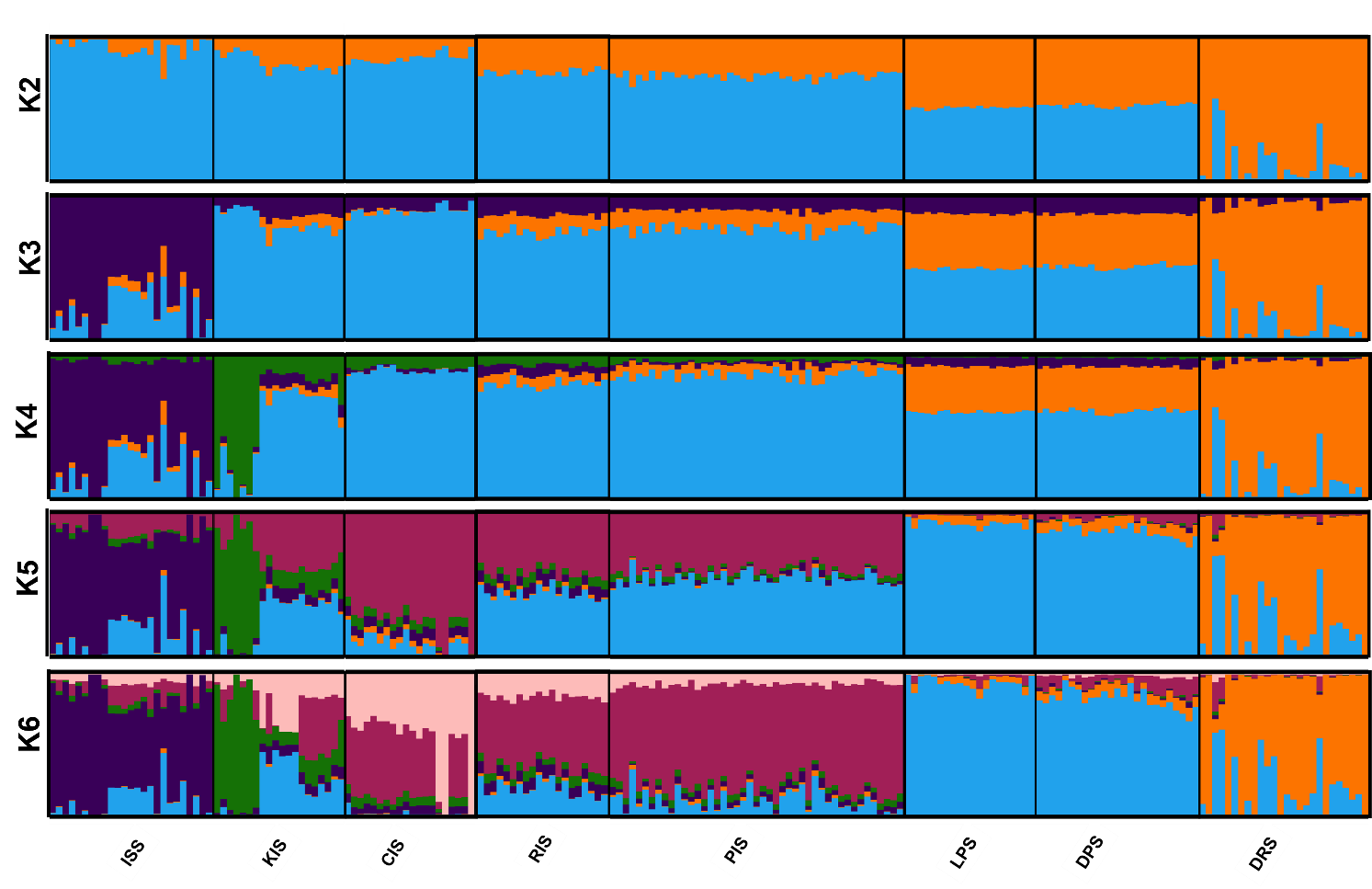


**Supplementary Figure 4.** Model-based clustering of Croatian sheep breeds. The analysis was performed using the program STRUCTURE, where K represents the assumed number of populations. Each breed is represented by a breed abbreviation: CIS – Cres Island Sheep, DPS – Dalmatian Pramenka, DRS – Dubrovnik Sheep, LPS – Lika Pramenka, PIS – Pag Island Sheep, RIS – Rab Island Sheep, KIS – Krk Island Sheep, ISS – Istrian Sheep
