# Supplementary material for: High-Density Genomic Characterization of Native Croatian Sheep Breeds
Source: Front Genet. 2022 Jul 15;13:940736. doi: 10.3389/fgene.2022.940736 (PMC9337876; doi:10.3389/fgene.2022.940736)
Supplement: Supplementary file 1 [file Presentation1.zip › Supplementary Figure 5.docx]

Supplementary Material

*
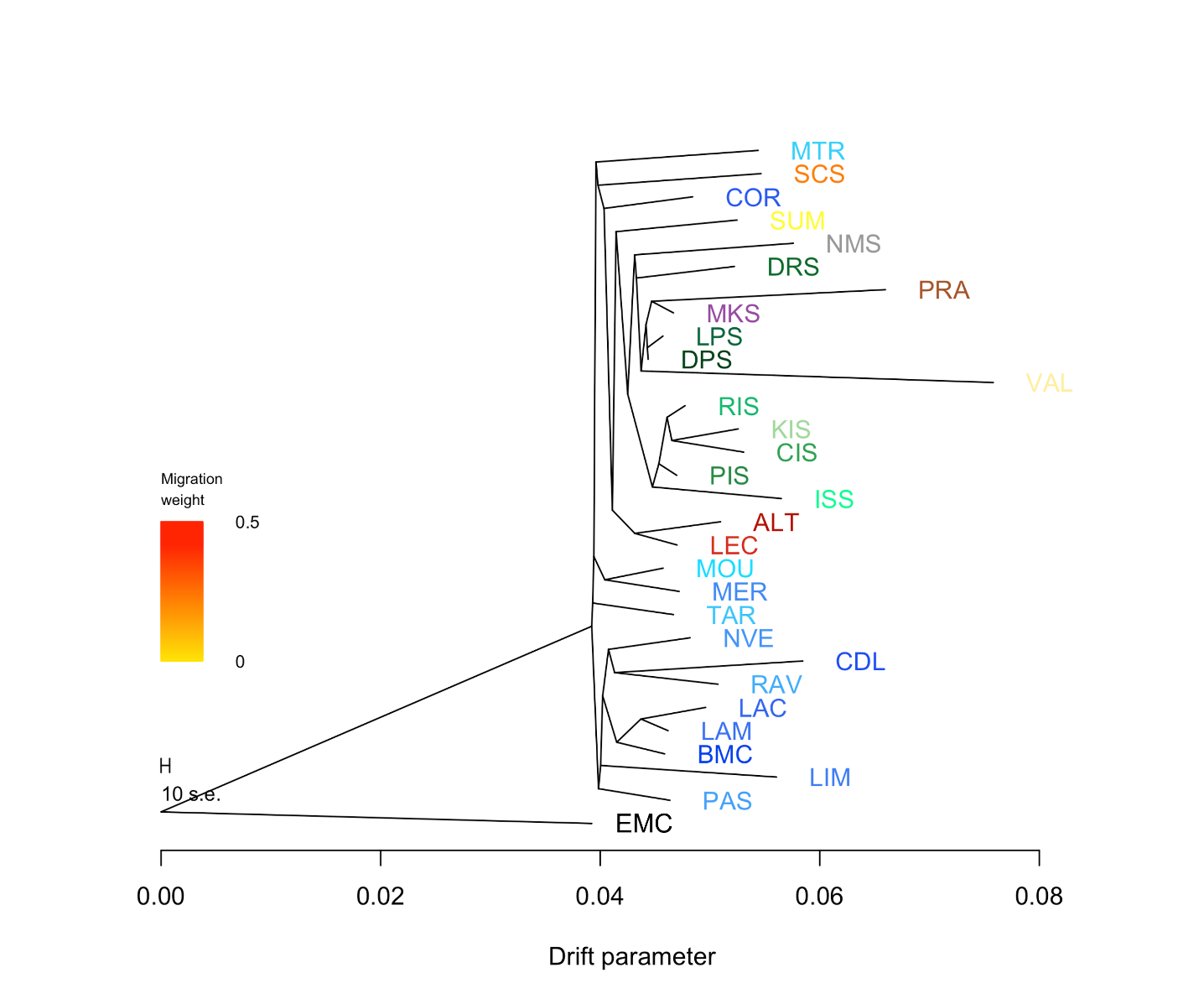
*

Supplementary Figure 5. Maximum likelihood tree for Mediterranean Sheep breeds, assuming no migration event. The tree was derived using TreeMix v.1.12. The European mouflon was set as the outgroup. Each country is represented in a different colour:: Croatia – green; France – blue; Spain – orange; Italy – red; North-Macedonia – grey; Ukraine – purple; Czech Republic – yellow; Serbia – brown. Within a country, each breed is represented with a different main colour and three-letter coding: DRS - Dubrovnik Sheep, LPS - Lika Pramenka, DPS - Dalmatian Pramenka, PIS - Pag Island Sheep, RIS - Rab Island Sheep, CIS - Cres Island Sheep, KIS - Krk Island Sheep, ISS - Istrian Sheep, PRA - Serbian Pramenka; VAL - Valachian; MKS - Mount Carpatian Sheep; NMS - North Macedonian Pramenka; SUM - Sumavaska; ALT - Altamunrana; LEC - Leccese; SCS - Churra; COR - Corse; MER - Merino; NVE - Noire du Velay; CDL - Causse du Lot; RAV - Rava; BMC - Blanche du Massif Central; LAM - Meat Lacaune; LAC - Milk Lacaune; LIM - Limousine; TAR - Tarasconnaise; MTR - Manech Tete Rouge; PAS - Preaples du Sud; MOU - Mourerous.
