# Supplementary material for: High-Density Genomic Characterization of Native Croatian Sheep Breeds
Source: Front Genet. 2022 Jul 15;13:940736. doi: 10.3389/fgene.2022.940736 (PMC9337876; doi:10.3389/fgene.2022.940736)

Supplementary Material

# Supplementary Data 1. Description of Croatian native sheep breeds

In Croatia there are nine native sheep breeds: Istrian sheep, Cres Island sheep, Rab Island sheep, Krk Island sheep, Pag Island sheep, Dalmatian Pramenka, Lika Pramenka, Cigaja and Dubrovnik sheep, which account for 75% of the total number of sheep in the Republic of Croatia. The Croatian sheep breeds belong to the Pramenka type or were created on the basis of the Pramenka type sheep.

Istrian Sheep

Istrian sheep are bred exclusively on the Istrian peninsula. There is no evidence of the origin of the Istrian sheep, but it is believed that originated on the basis of northern Italian breeds, especially the Bergamo sheep. It looks quite different from the other Croatian native sheep breeds. It can be recognized by its convex nose profile, black and white wool and hair color, and developed horns. The Istrian sheep has a larger build and reaches an average body weight of 70 kg. According to the production type, it belongs to the group of dairy sheep and is the most milk-producing Croatian native sheep breed. It produces an average of 135-145 liters of milk during lactation (6-8 months).


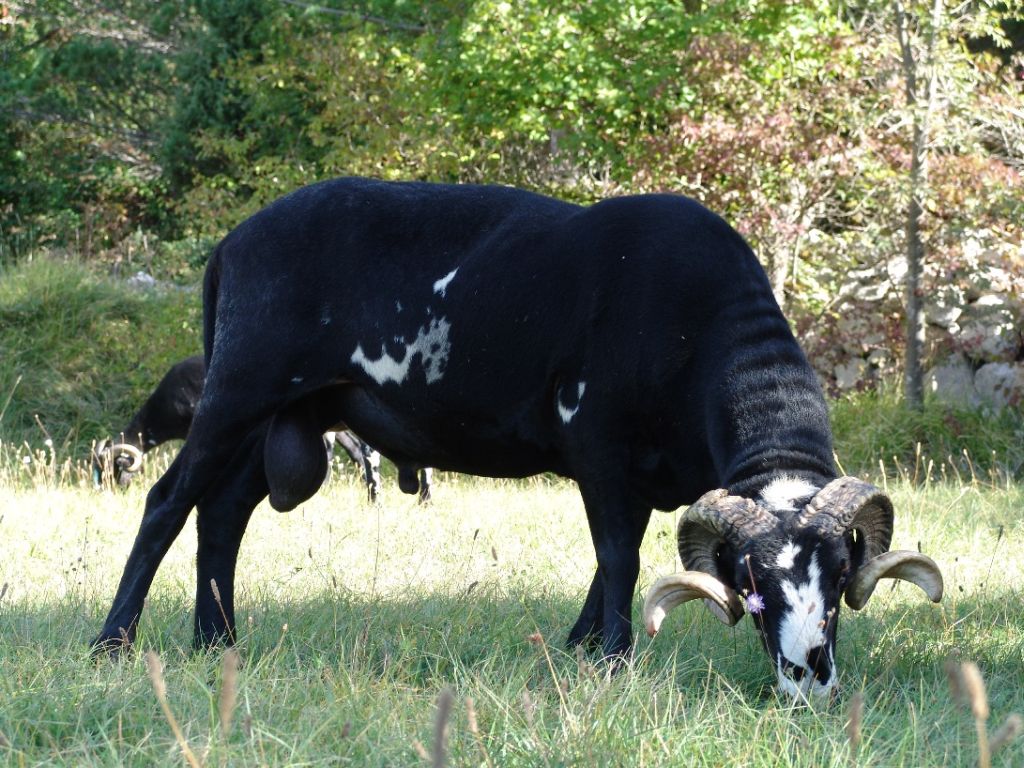


Cres Island Sheep

Cres Island Sheep are bred today on the islands of Cres and Lošinj. There is no exact information about the origin of Cres sheep. The first records of Cres Island Sheep date back to 1332, where sheep breeding is mentioned as an important economic activity on the island of Cres. Between the two world wars, crossbreeding between Cres Island Sheep and Karakul rams was carried out in order to produce furs. After the World War II local sheep were crossed with merino rams of the Gentile di Puglia breed, but the crosses were not used for further breeding. The Cres Island Sheep has a smaller body of about 45 kg. Its body is covered with a semi-closed white fleece, while its legs and belly are covered with hair.


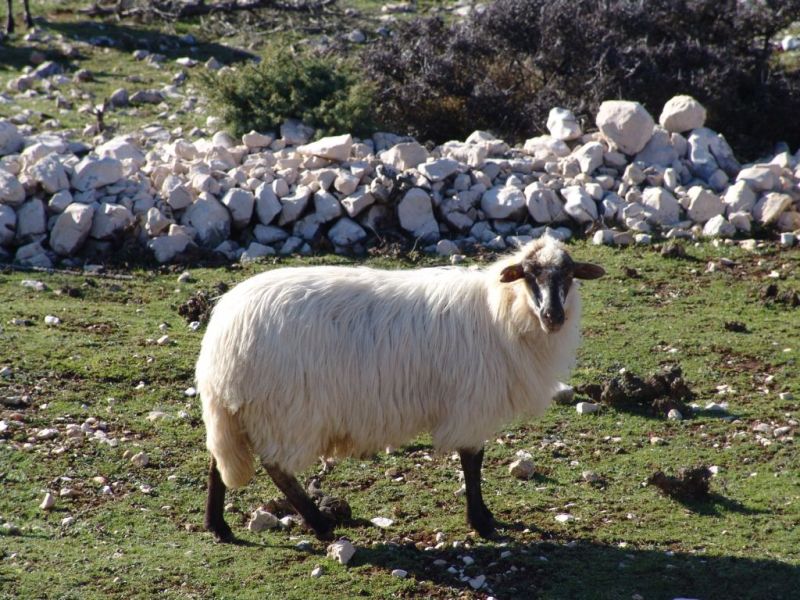


Rab Island Sheep

The Croatian native sheep, which was and is bred exclusively on the island of Rab. Although it is believed, based on the phenotypic characteristics that merino sheep had an influence on the origin of the breed, there are no records of this. Today's breeding is represented by the old island sheep type and it is believed that there were no genetic influences from other breeds for decades. The breed is characterized by hardiness, humility and adaptability to rough terrain, hence the local name "škraparica". It belongs to a group of sheep with combined production traits, where the emphasis is on meat. The Rab Island Sheep is a small and robust sheep weighing between 30 and 45 kg. The body is covered with semi-closed white fleece.


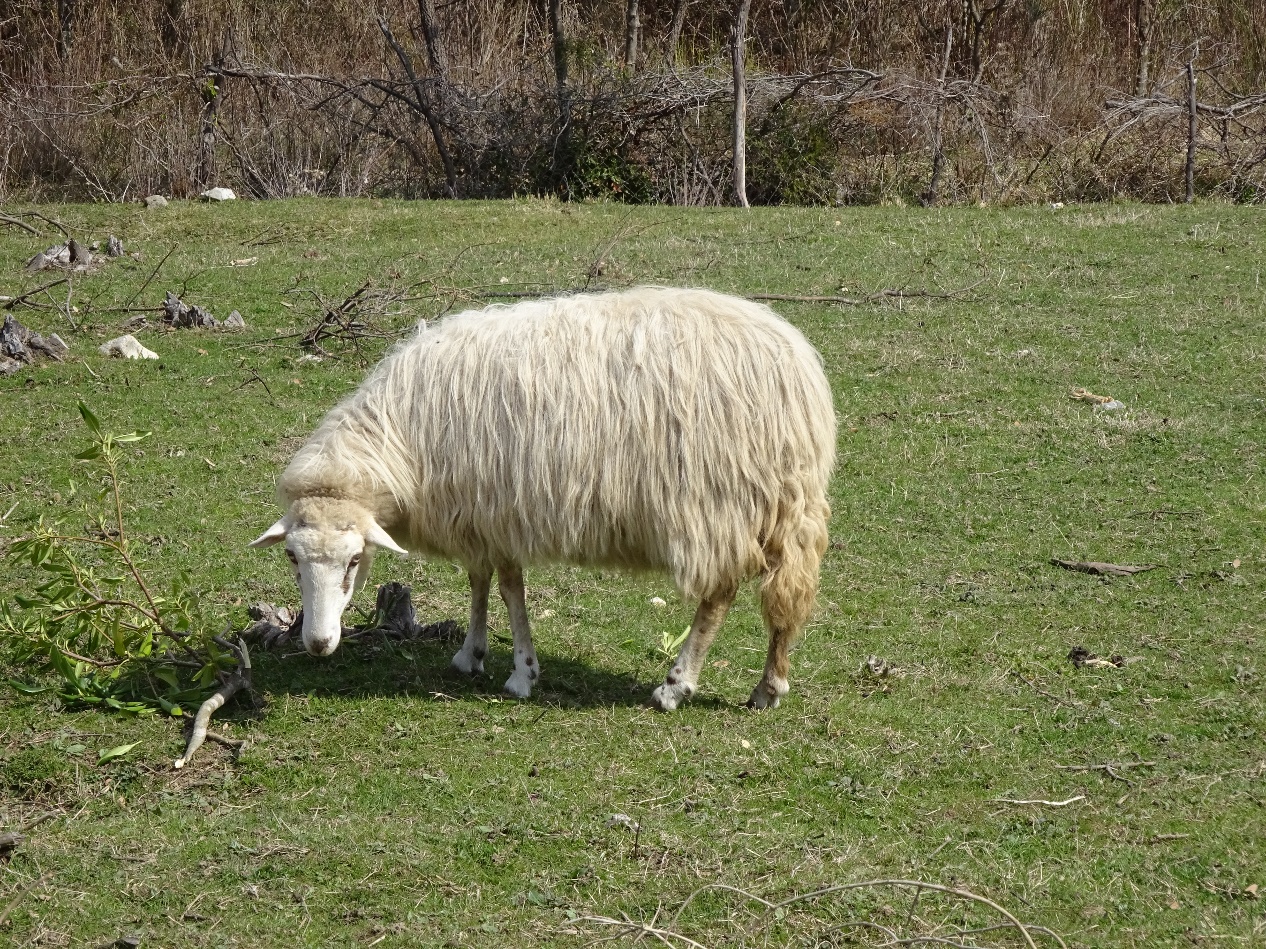


Krk Island Sheep

The Krk Island Sheep was created by crossing local sheep on the island of Krk with imported Merino sheep. The exact date of the breed's origin is not known, but the first records of the breed date back to the 17th century, when Venetian reports mention that a total of 34,740 small ruminants were bred on the island of Krk. The Krk Island Sheep is an adaptable, hardy, modest sheep with a smaller body and a weight of about 35 kg. It belongs to the group of sheep with combined production characteristics, with emphasis on meat. The wool of most animals is white with a semi-closed type, although there are individuals with brown, gray and black fleece. The belly and lower legs are not covered with wool, but with dense hair.


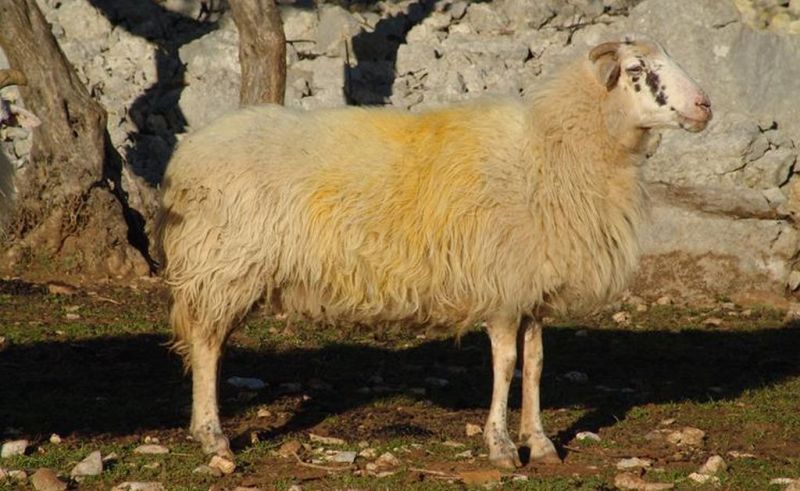


Pag Island Sheep

The Pag Island Sheep was created by crossing the original island breed with the merino rams of the Negretti and Bergamo breeds. Besides these breeds, the influence of the Gentile di Puglia breed is also mentioned. Work on the genetic improvement of the Pag Island Sheep began in 1870 with the establishment of the Society for the Improvement of Sheep Breeding "Gregge Modella". In the second half of the 20th century, there were attempts to breed the Pag Island Sheep with Sardinian and Awassi breeds to increase milk yield, but environmental conditions did not allow these breeds to be kept in the Pag Island Sheep genotype. The Pag Island Sheep is primarily intended for milk production. The most important products are the world famous Pag cheese and Pag lamb meat. The body of a the Pag Island Sheep is covered with a closed to semi-closed white fleece, while the belly and legs are covered with hair that has freckles of various sizes and colors. The body weight of the sheep is about 45 kg.


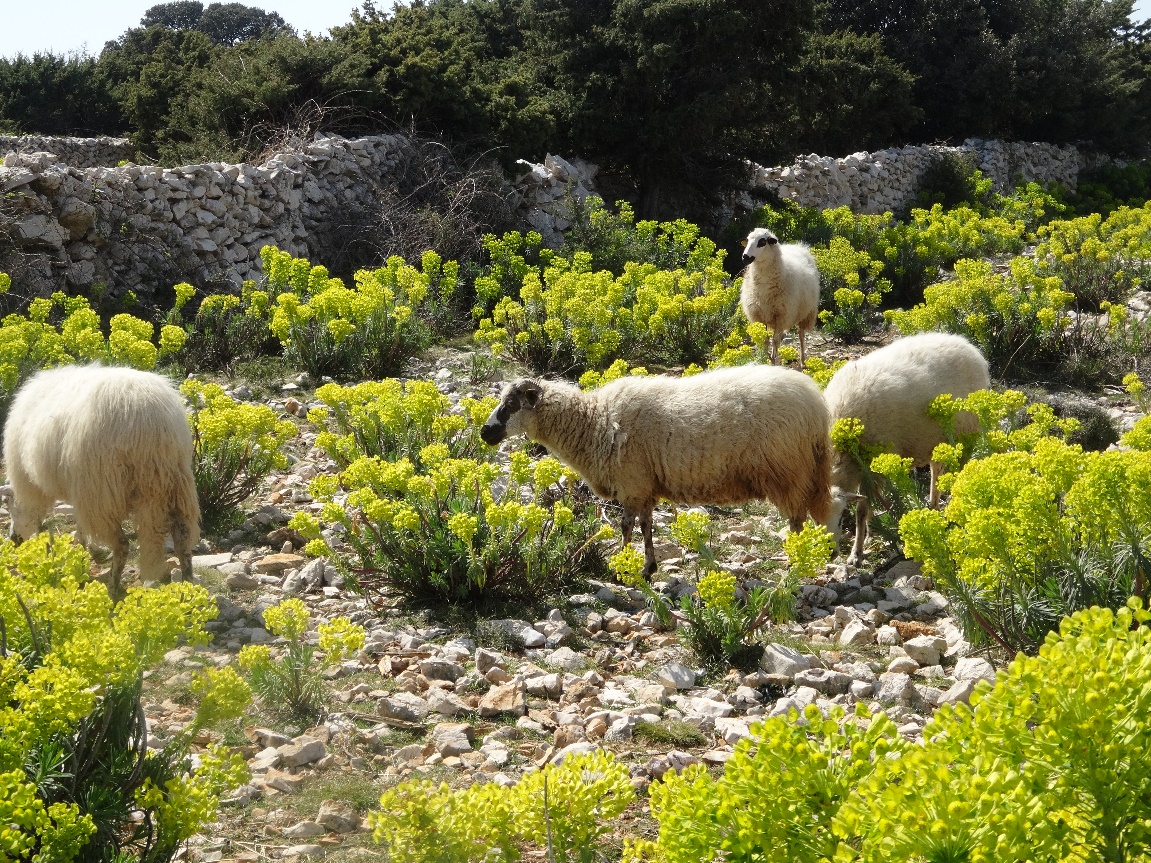


Dalmatian Pramenka

Dalmatian Pramenka includes native sheep bred in the Velebit, Dinara, Svilaja, Kamešnica, Biokovo, Dalmatian hinterland, Ravni kotari, Cetinje region and on some islands. Since the breed was defined only recently, there are no records of its origin. It is believed that the origin of the breed is closely related to other Pramenka breds in the Balkan Peninsula. The Dalmatian Pramenka is a small sheep with a strong constitution. Although the combined production traits are mainly used for meat production. Their body is covered with an open fleece, mostly white. The head, like the legs, is usually black pigmented, although there are animals with white heads.


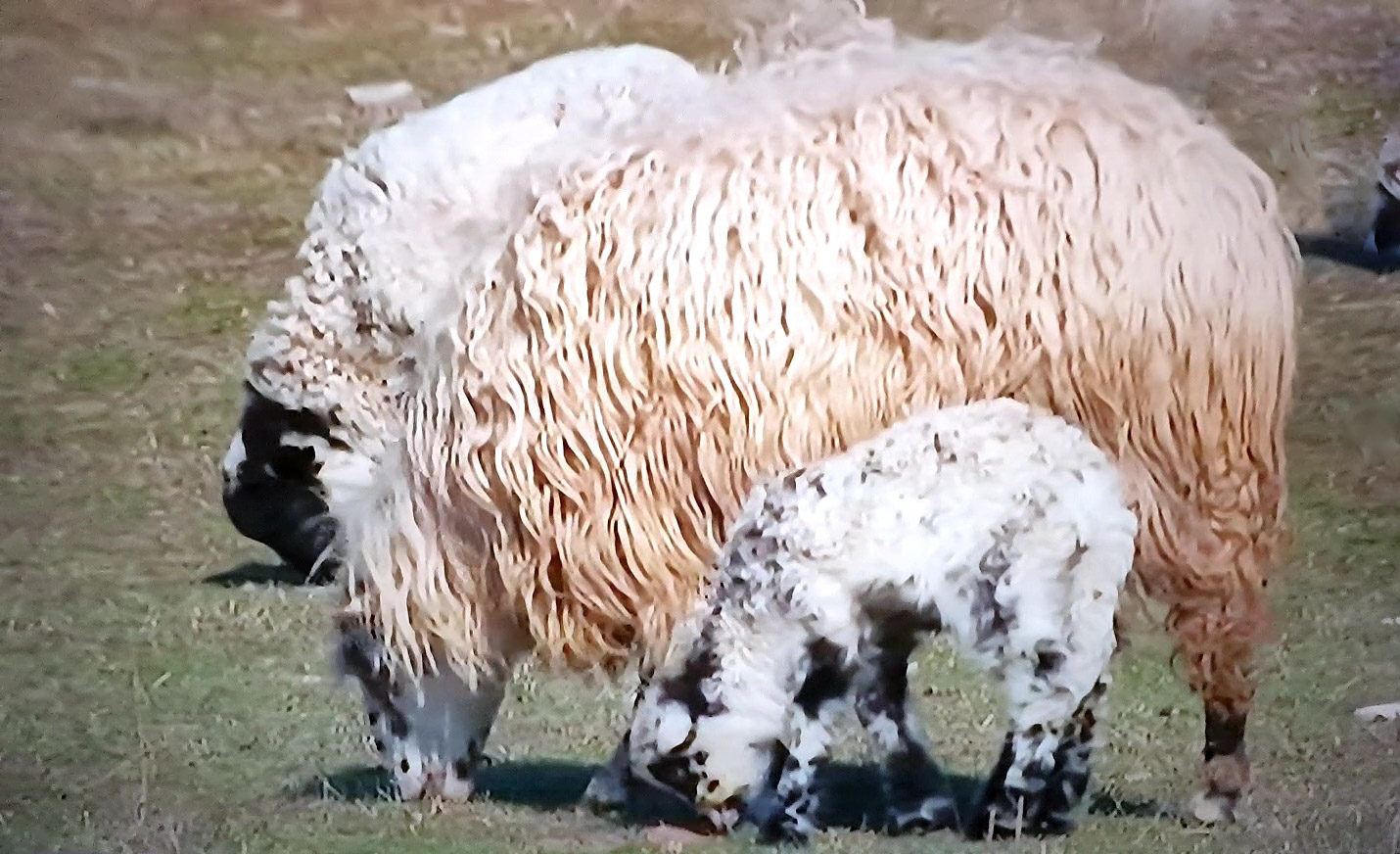


Lika Pramenka

The Lika Pramenka originates from the mountain regions of Lika and Gorski Kotar. The breed is particularly adapted to harsh winters and dry summers. Throughout history there have been breed improvement attempts, first with rams of the Dubska and Privorska Pramenka breeds, and later with the European meat breeds Ille de France, Merinolandschaf and some Australian breeds. The body of Lika Pramenka is covered with open white fleece consisting of lacy and whip-like strands, while the head, legs and belly are covered with short, mostly darker hair. Lika Pramenka is a medium-sized sheep with a harmonious and firm body and a weight of about 50 kg.


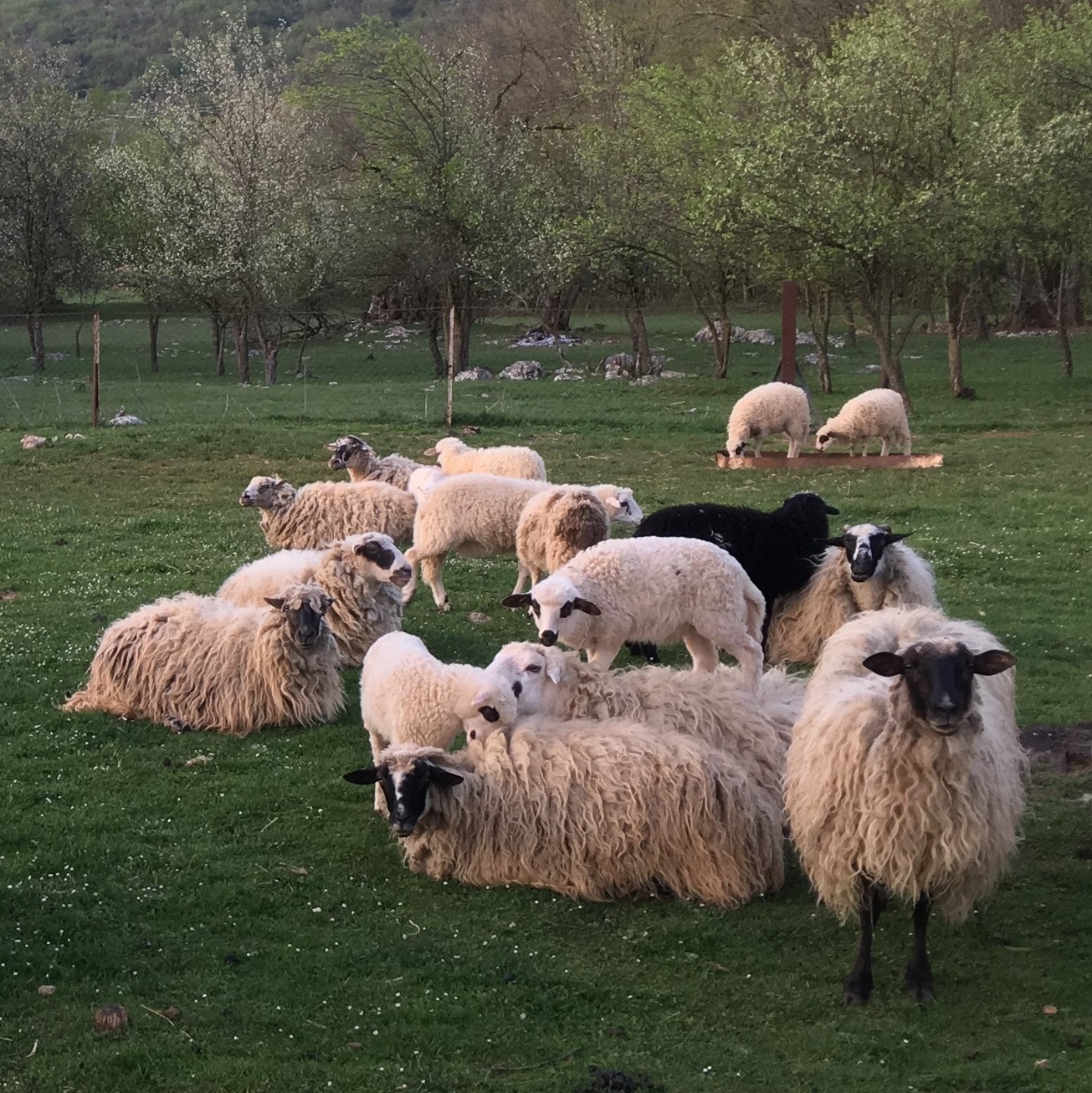


Dubrovnik Sheep

The place and time of origin of the Dubrovnik sheep are not known, but it is believed to have resulted from the crossing of the native Pramenka with the Merino breed during the Dubrovnik Republic. Dubrovnik sheep are bred along a narrow Adriatic coast from the Pelješac peninsula to the border with Montenegro, mainly around Imotica and in Konavle. The Dubrovnik sheep is a medium-sized, harmoniously built sheep with a weight of about 50 kg. Its body is covered with a closed to semi-closed white fleece and its head, belly and legs are covered with dense hair. During the Homeland War and the occupation of Dubrovnik-Neretva County, the number of heads decreased drastically and the breed almost disappeared. Through the joint work of several institutions, the breed was revived in 2005-2006, but it is still our most endangered breed.


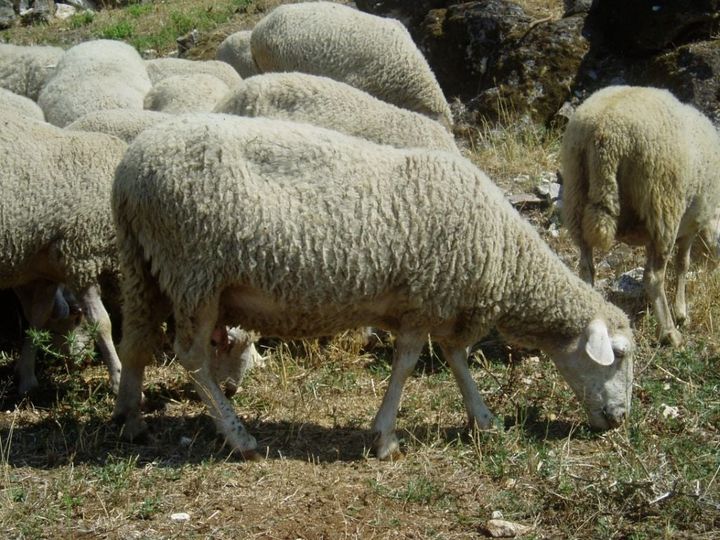

Supplement: Supplementary file 3 [file DataSheet1.docx]
